# ENT1 and treatment of viral diseases

**DOI:** 10.18632/oncotarget.5859

**Published:** 2015-09-28

**Authors:** Rajesh Panigrahi, Srikanta Dash

**Affiliations:** Department of Pathology and Laboratory Medicine, Tulane University Health Sciences Center, New Orleans, LA, USA

**Keywords:** autophagy, ENT1

**Figure 1:**
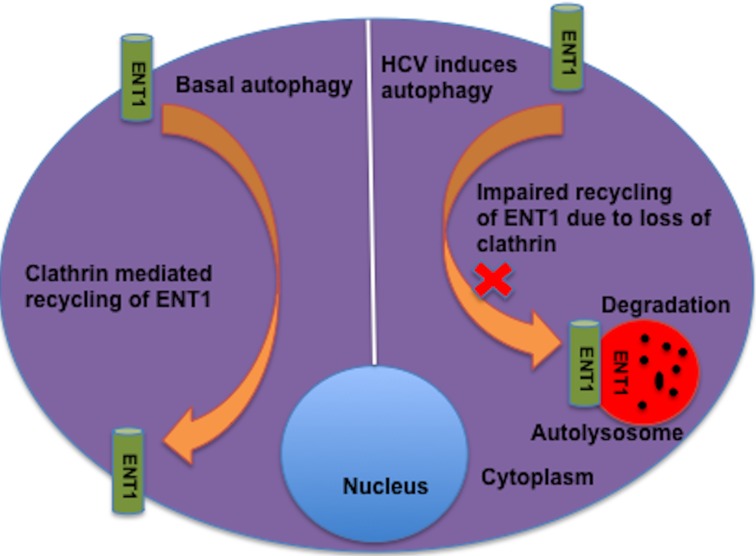
Diagram summarizing how HCV induced autophagy response decreases the cell surface expression of nucleoside transporter-1 (ENT-1) through clathrin mediated recycling. Loss of clathrin heavy chain due to increased autophagy response impairs recycling of ENT1 forcing it for degradation in the autolysosome.

Ribavirin (RBV) is a synthetic nucleoside analogue (1-b-D-ribofuranosyl-1, 2,4-triazole-3-carboxamide) used for the treatment of a number of viral diseases. RBV inhibits inosine 5′-phosphate dehydrogenase (IMPDH), the enzyme required for synthesis of guanosine monophosphate (GMS) as well as DNA and RNA synthesis. Based on this mechanism of action, RBV is also used in the treatment of solid tumors, lymphoproliferative disease and some inflammatory diseases such as Crohn's disease. A number of nucleoside analogs including RBV diffuse across cell membranes through a membrane transporter protein called equilibrative nucleoside transporter-1 (ENT1). Our recent publication demonstrated that an autophagy response induced by hepatitis C virus (HCV) in a cell culture reduces RBV uptake and antiviral activity by diminishing the surface expression of ENT1 [1]. Moreover, our findings provide a potential mechanism explaining how the expression of ENT1 is modulated by clathrin heavy chain when cellular autophagy response is increased. We showed that expression level of clathrin heavy chain is decreased due to increased autophagy response. Down regulation of clathrin heavy chain by autophagy prevents ENT1 recycling to the plasma membrane, forcing ENT1 to the lysosome for degradation (Figure 1). We also showed that autophagy induction by the small molecule inhibitor Torin 1 decreases the expression of ENT1 on cell surface, indicating that an increased autophagy response due to viral and non-viral causes could modulate the expression of ENT1 on cell membranes. We found that RBV antiviral activity against HCV is enhanced when the cellular autophagy response inhibited by using hydroxychloroquine. It is important to mention that this study will have broader implications for understanding the treatment response of other viral diseases and cancers by using nucleoside analogues as a drug target. We discussed how this finding could be explored to improve the therapeutic response of RBV against viral disease and cancer by inhibiting cellular autophagy.

Human ENTs are the nucleoside transporter proteins expressed on the cell membrane that occur as four isoforms (ENT1-ENT4). These proteins play important roles in transporting nucleosides- and nucleotides-based small molecule drugs. The expression of ENT1 is critical for salvage of natural nucleotides and nucleosides for nucleic acid synthesis, neurotransmission, and regulation of cardiovascular activity [2]. The expression of ENT1 is used as a biomarker in the clinic for uptake of exogenous nucleoside and nucleotide analogues used for the treatment of viral disease and cancer. The ENT1 mRNA is translated and protein is folded in the endoplasmic reticulum (ER) then transported via Golgi apparatus to the plasma membrane. In the plasma membrane the expression of ENT1 is maintained by internalization and recycling through clathrin-mediated endocytosis. The membrane expression of ENT1 is linked to cellular endocytosis and expression of the clathrin heavy chain. Recently two publications demonstrated that clathrin heavy chain is directly involved in the formation of autophagolysosomes and autophagic lysosme reformation [3, 4]. These invstigators showed that silencing clathrin heavy chain decreases autophagolysosome formation. The clathrin heavy chain level is decreased when cellular autophagy is increased [1]. Autophagy is a highly conserved, lysosome based degradation process. Since the cellular autophagy response is increased in viral infection, cancer and also during chemotherapy, we anticipate that the proposed autophagy related mechanism that modulates the ENT1 expression on cell surface could play an important role in drug response.

Taking into consideration the previously mentioned autophagy related mechanism that regulates the expression of ENT1, it is reasonable to speculate that cellular autophagy status could regulate the uptake and response to nucleoside based treatment of viral infection or cancer. RBV has been used for the treatment of a number of viral infections with modest antiviral activity. The decreased antiviral activity of RBV against virus disease could be related to the degree of cellular autophagy response and expression of ENT1. Based on our results, we propose that inhibiting cellular autophagy response should improve the RBV uptake and antiviral activity against other viruses. One report showed that RBV has a potent antitumor activity against metastatic breast cancer [5]. The cellular uptake of a number of nucleotide analogs such as mercaptopurine cytarabine, fludarabine, pentostatin, cladribine, azacitinine, clofarabine, nelarabine, decitabine, floxuridine, gemcitabine, capacitabine and 5-fluorouracil (used for the treatment of lymphoproliferative disease, solid tumors and pancreatic cancer) may be inflammed by ENT1 expression in cancer cells. All of the above nucleosides enter to cancer cells primarily via ENT1 are metabolized intracellularly and incorporated into DNA and RNA resulting in chain termination, apoptosis and cell death. We also anticipate that the treatment of some other cancers such as lymphoma and pancreatic cancer by nucleoside analogs could be affected by the reduced ENT1 expression by autophagy. We postulate that decreased expression of ENT1 due to increased autophagy response could have a negative impact on chemotherapy response of the above described nucleoside analogs.

The responses to gemcitabine treatment of pancreatic cancer have been shown to be correlate with reduced expression of ENT1 transporter. Over expression of ENT1 enhances the gemcitabine response in human pancreatic cancer, tumor cells lacking ENT1 expression are highly resistant to gemcitabine [2]. This is also supported by a number of clinical studies showing that impaired expression of ENT1 correlates with drug resistance in non-small cell lung cancer [2]. Significant correlation has also been found between gemcitabine chemotherapy and ENT1 expression in the treatment of bladder cancer [2]. It remains to be tested whether inhibiting autophagy response could restore ENT1 expression and improve gemcitabine chemotherapy. In summary, our results, as well as the hypothesis presented herein related to cellular autophagy regulation of clathrin and ENT1 membrane expression will shed light on the clinical implication and mechanism of treating of viral infection and cancer with nucleoside analogs.
